# *Borrelia* Diversity and Co-infection with Other Tick Borne Pathogens in Ticks

**DOI:** 10.3389/fcimb.2017.00036

**Published:** 2017-02-14

**Authors:** Cristian Raileanu, Sara Moutailler, Ionuţ Pavel, Daniela Porea, Andrei D. Mihalca, Gheorghe Savuta, Muriel Vayssier-Taussat

**Affiliations:** ^1^INRA, UMR Bipar, INRA, Anses, ENVAMaisons-Alfort, France; ^2^Department of Public Health, Faculty of Veterinary Medicine, University of Agricultural Sciences and Veterinary MedicineIaşi, Romania; ^3^Department of Parasitology and Parasitic Diseases, University of Agricultural Sciences and Veterinary MedicineCluj-Napoca, Romania

**Keywords:** questing ticks, *Borrelia*, tick-borne pathogens, co-infection, Romania

## Abstract

Identifying *Borrelia burgdorferi* as the causative agent of Lyme disease in 1981 was a watershed moment in understanding the major impact that tick-borne zoonoses can have on public health worldwide, particularly in Europe and the USA. The medical importance of tick-borne diseases has long since been acknowledged, yet little is known regarding the occurrence of emerging tick-borne pathogens such as *Borrelia* spp., *Anaplasma phagocytophilum, Rickettsia* spp., *Bartonella* spp., “*Candidatus* Neoehrlichia mikurensis”, and tick-borne encephalitis virus in questing ticks in Romania, a gateway into Europe. The objective of our study was to identify the infection and co-infection rates of different *Borrelia* genospecies along with other tick-borne pathogens in questing ticks collected from three geographically distinct areas in eastern Romania. We collected 557 questing adult and nymph ticks of three different species (534 *Ixodes ricinus*, 19 *Haemaphysalis punctata*, and 4 *Dermacentor reticulatus*) from three areas in Romania. We analyzed ticks individually for the presence of eight different *Borrelia* genospecies with high-throughput real-time PCR. Ticks with *Borrelia* were then tested for possible co-infections with *A. phagocytophilum, Rickettsia* spp., *Bartonella* spp., “*Candidatus* Neoehrlichia mikurensis”, and tick-borne encephalitis virus. *Borrelia* spp. was detected in *I. ricinus* ticks from all sampling areas, with global prevalence rates of 25.8%. All eight *Borrelia* genospecies were detected in *I. ricinus* ticks: *Borrelia garinii* (14.8%), *B. afzelii* (8.8%), *B. valaisiana* (5.1%), *B. lusitaniae* (4.9%), *B. miyamotoi* (0.9%), *B. burgdorferi* s.s (0.4%), and *B. bissettii* (0.2%). Regarding pathogen co-infection 64.5% of infected *I. ricinus* were positive for more than one pathogen. Associations between different *Borrelia* genospecies were detected in 9.7% of ticks, and 6.9% of *I. ricinus* ticks tested positive for co-infection of *Borrelia* spp. with other tick-borne pathogens. The most common association was between *B. garinii* and *B. afzelii* (4.3%), followed by *B. garinii* and *B. lusitaniae* (3.0%). The most frequent dual co-infections were between *Borrelia* spp. and *Rickettsia* spp., (1.3%), and between *Borrelia* spp. and “*Candidatus* Neoehrlichia mikurensis” (1.3%). The diversity of tick-borne pathogens detected in this study and the frequency of co-infections should influence all infection risk evaluations following a tick bite.

## Introduction

In Europe, ticks are the most ecologically important vectors of pathogens that cause both human and animal diseases (de la Fuente et al., 2008). *Ixodes ricinus* ticks are the most common ticks in Europe, and are broadly distributed across the entire continent. They are capable of transmitting a wide variety of zoonotic pathogens, such as viruses, bacteria, protozoa, and even helminths (Egyed et al., 2012). *I. ricinus* is also the most common questing tick species in Romania, representing 86.9% of ticks, followed by *Dermacentor marginatus* (9.5%), *Haemaphysalis punctata* (2.6%), and *D. reticulatus* (0.02%) (Mihalca et al., 2012a). Because *I. ricinus* feeds on a broad range of animals, this particular tick species transmits the widest variety of pathogens, including bacteria, parasites, and viruses. Amongst all *I. ricinus*-borne diseases reported in Europe, Lyme disease caused by some genospecies of *Borrelia burgdorferi* sensu lato (s.l.), is by far the most prevalent, with an estimated 85,000 annual cases (Subramanian et al., 2012). *B. burgdorferi* s.l. group currently comprises over 20 genospecies, nine of which are circulating in Europe (Clark et al., 2014). Among these, five genospecies are human pathogens: *B*. *afzelii, B. garinii, B. burgdorferi* sensu stricto (s.s.), *B. bavariensis*, and *B. spielmanii;* three are suspected human pathogens: *B*. *lusitaniae, B. valaisiana*, and *B. finlandensis;* and one genospecies, *B. bissettii*, has no clinical relevance to humans. Another *Borrelia* genospecies, *Borrelia miyamotoi*, belongs to the relapsing fever group and is transmitted by the same *Ixodes* species that also transmits *B. burgdorferi* s.l.. In 2013 *B. miyamotoi* was identified as a human pathogen in Europe (Hovius et al., 2013). This bacterium has been also recently identified in Romania (Kalmár et al., 2016).

The same tick may be co-infected with different *Borrelia* genospecies (Cosson et al., 2014) and various other tick-borne pathogens, with the possibility of co-transmission to either humans or animals (Moutailler et al., 2016). These co-infections might have consequences in terms of pathogen co-transmission (Swanson et al., 2006; Nieto and Foley, 2009; Agoulon et al., 2012; Chowdri et al., 2013; Gugliotta et al., 2013; Horowitz et al., 2013; Hovius et al., 2013; Tijsse-Klasen et al., 2013; Cosson et al., 2014; Knapp and Rice, 2015) that may also have important implications and relevance to public health (Diuk-Wasser et al., 2016). Indeed, co-infection in humans and animals might enhance disease severity as has been reported for concurrent babesiosis and Lyme disease (Grunwaldt et al., 1983; Golightly et al., 1989), or may have consequences in term of treatment and diagnosis (Diuk-Wasser et al., 2016).

The geopolitical location of Romania at the eastern border of the EU is strategically important from an epidemiological point of view. It represents a continual risk for emerging disease, not only in Romania, but also as a gateway into Europe. Only partial data has been collected on the prevalence of the Lyme disease agent or other tick-borne pathogens in eastern Romanian ticks and wildlife. However, it has been shown that Lyme disease is endemic in southern, eastern, and southeastern parts of Romania (Kalmar et al., 2013). From 2010, the Romanian National Centre for Surveillance and Control of Communicable Diseases has issued annual reports detailing the epidemiological situation of Lyme disease in humans. An average of 340 confirmed human Lyme disease cases were recorded annually between 2010 and 2015, with a mean incidence rate of 2.1 per 100,000 population, being situated at the lower level of the European incidence that ranges per country from less than one per 100,000 population to about 350 per 100,000 population (Rizzoli et al., 2011). Several studies have already reported the presence of *Borrelia* genospecies in questing ticks in Romania. Interestingly, these studies identified *B. burgdorferi* s.l. in *I. ricinus* ticks at a lower prevalence compared to the European average of 13.6% (Rauter and Hartung, 2005). A study involving over 12,000 questing ticks collected from 183 localities across all Romanian counties reported an overall *B. burgdorferi* s.l. prevalence of 1.4% (Kalmar et al., 2013). An alternate study of three southern and central Romanian counties recorded Lyme disease agent prevalence of up to 18.0% in unfed ticks (Coipan and Vladimirescu, 2011). Other studies in Romania have identified *B. burgdorferi* s.l. in *I. ricinus* ticks collected from different hosts such as lizards (Majláthová et al., 2008), horses (Ionita et al., 2013), hedgehogs (Dumitrache et al., 2013), and humans (Briciu et al., 2014). There are also reports of *B. burgdorferi* s.l. in tissues of wild mustelids (Gherman et al., 2012) and red foxes (Dumitrache et al., 2015). In these studies, the most commonly identified genospecies were *B. afzelii, B. garinii*, and *B. burgdorferi* s.s.

In Romania, as elsewhere in the world, limited work has been carried out on the occurrence of different *Borrelia* genospecies co-infections in questing ticks, including *Borrelia* with other tick-borne pathogens. Indeed, most studies have focused on the identification of single or a specific limited numbers of pathogens (Ionita et al., 2013, 2016; Kalmar et al., 2013; Matei et al., 2015; Kalmár et al., 2016), mainly due to technical constraints. Recently, we have implemented efficient high-throughput methods that enable broad-range co-detection of the most important known or putative tick-borne pathogens (*Borrelia* spp., *Rickettsia* spp., *A. phagocytophilum*, “*Candidatus* Neoehrlichia mikurensis”, *Babesia* spp., *Bartonella* spp. etc.) in individual tick samples (Michelet et al., 2014).

In the present study, our objective was to identify (co-) infection rates for different *Borrelia* genospecies along with other tick-borne pathogens in questing ticks collected from three sites in eastern Romania: One urban region dedicated to recreational activities and two forested areas.

## Materials and methods

### Tick sampling

**Figure 1:**
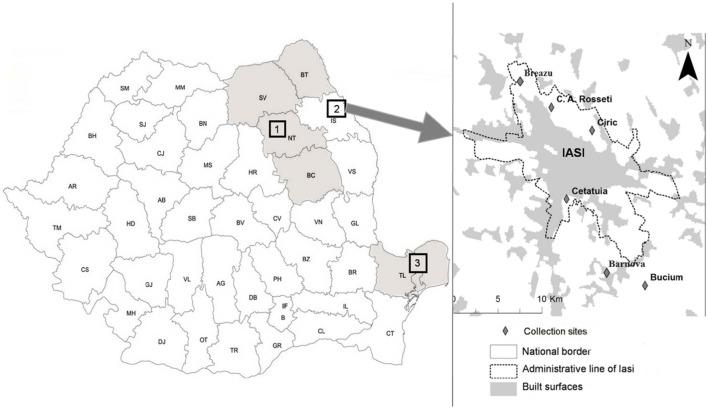
**Sampling areas in eastern Romania**. Geographical areas were located in eastern Romania, comprising six counties across three distinct zones. Sampling areas 1 and 3 were mainly forested habitats, whereas sampling area 2 included six sites located in the vicinity of Iaşi recreational areas (C.A. Rosetti, Breazu, Ciric, Cetăţuia, Bârnova, and Bucium); Tick sampling took place from May 2013 until September 2014. (1)—Sampling area 1 covering four counties: SV, Suceava; BT, Botoşani; NT, Neamt; BC, Bacău.(2)—Sampling area 2: Iaşi—representing collection sites located in Iaşi recreational areas (Breazu, C.A. Rosseti, Ciric, Cetăţuia, Bârnova, Bucium). (3)—Sampling area 3 located in Tulcea county.

Questing ticks were collected from 19 sampling sites distributed across six counties, covering three geographically distinct areas of eastern Romania (Figure 1). Each area represented a distinct habitat: A forested area in the north-eastern part of the country (sampling area 1), suburban sites intended for recreational activities (sampling area 2), and sites located in the southern part of the country including a forested and an arid region (sampling area 3). Ticks were collected by the dragging method. Dragging was performed over 5 transects of 300 m at each location. Each transect was divided into 10 sub-transects which were separated by 20 m, and the flag was examined after every 10 m of dragging, at the end of each segment. The surface covered at each collection site was ~1.5 hectares. In sampling area 2 (Figure 1), monthly collection was performed at each site, from March until September 2014. Tick collection in areas 1 and 3 was performed only once at each site, between May 2013 and September 2014.

Sampling area 1 included six forested collection sites across four counties that were selected based on their proximity to rural settlements. (46.536747 N, 26.821123 E; 47.23221 N, 26.54634 E; 46.517207 N, 26.187067 E; 47.780000 N, 25.718600 E; 47.550000 N, 25.950000 E; and 47.779500 N, 26.516000 E) (Figure 1).

Sampling area 2 included six collection sites near Iaşi city recreational areas [Breazu (47.212044 N, 27.530304 E), C.A. Rosetti (47.204710 N, 27.556685 E), Ciric (47.178178 N, 27.609791 E), Cetăţuia (47.132564 N, 27.582936 E), Bârnova (47.059598 N, 27.640815 E), and Bucium (47.068330 N, 27.664561 E)] (Figure 1). The main criterion used for selection of collection sites was potential public exposure to vector ticks, and thus we included the major recreational sites around the city of Iaşi. Deciduous trees and meadows are the predominant vegetation at these sites (designated suburban forest) (Pavel et al., 2014).

Sampling area 3 included seven collection sites from Tulcea County (45.269722 N, 28.459722 E; 45.215936 N, 28.415648 E; 45.26293 N, 28.16968 E; 44.891311 N, 28.717223 E; 44.873796 N, 28.735763 E; 44.865767 N, 28.810092 E and 44.86309 N, 28.689242 E) that were located mostly in forested and arid zones (Figure 1). This area has an arid or coastline type climate (Moise and Dumitru, 2012).

Tick species and developmental stages were identified under a stereomicroscope using standard morphological identification keys (Perez-Eid, 2007), then stored individually at −80°C.

### DNA and RNA extraction

Individual adult and nymph *I. ricinus* were lysed using 2.8 mm stainless steel beads in 300 μl of culture cell medium (Dulbecco's Modified Eagle medium, Gibco by Life technologies, UK) complemented with 10% fetal calf serum as previously described (Vayssier-Taussat et al., 2012). Ticks were homogenized using Precellys 24 lyser/homogenizer (Bertin, France) at 5500 rpm for 20 s. After homogenization, each tube was centrifuged at 1,500 rpm for 2 min. DNA and RNA extraction was performed on 100 μL aliquots using the Nucleospin Tissue kit or the Nucleospin RNA II kit (Macherey Nagel, Germany) according to the manufacturer's instructions. Purified DNA and RNA was eluted into 50 μL elution buffer, RNase-free water respectively.

### Detection of borrelia genospecies DNA in questing *I. ricinus*

The BioMark™ real-time PCR system (Fluidigm, USA) was used for high-throughput microfluidic real-time PCR for the detection of the genus *Borrelia*: Seven genospecies belonging to the Lyme disease spirochete group, *B. burgdorferi* s.l. (*B. burgdorferi* s.s., *B. afzelii, B. garinii, B. spielmanii, B. valaisiana, B. lusitaniae*, and *B. bissettii*) and one genospecies from the recurrent fever group (*B. miyamotoi*) (Table 1). A DNA pre-amplification step was performed in a final volume of 5 μL containing 2.5 μL TaqMan PreAmp Master Mix (2X), 1.2 μL of the pooled primer mix (0.2X mix containing specific primer pairs for each of the eight *Borrelia* genospecies analyzed) and 1.3 μL of tick DNA, with one cycle at 95°C for 10 min, 14 cycles at 95°C for 15 s and 4 min at 60°C. Following pre-amplification, qPCRs were performed using FAM- and black hole quencher (BHQ1)-labeled TaqMan probes (Michelet et al., 2014) with TaqMan Gene Expression Master Mix in accordance with manufacturer's instructions (Applied Biosystems, France). Thermal cycling conditions were as follows: 95°C for 5 min, 45 cycles at 95°C for 10 s, 60°C for 15 s, and 40°C for 10 s. Data were acquired on the BioMark™ real-time PCR system and analyzed using the Fluidigm real-time PCR Analysis software to obtain crossing point (CP) values. Assays were performed in duplicate and two negative water controls were included per analysis and none of them were found positive after amplification. *I. ricinus* DNA served to confirm the tested tick species and as a DNA extraction control (Michelet et al., 2014). Moreover, to determine if factors present in the samples could inhibit PCR (false negative results), *E. coli* strain EDC933 DNA was added to each sample as internal inhibition control (Michelet et al., 2014). *E. coli* and *I. ricinus* DNA were amplified in all samples confirming the absence of false negative results.

**Table 1:** **Primer sets for pathogen DNA amplification and sequencing in ticks**.

**Pathogen**	**Target gene**	**Primers**	**Nucleotide sequence (5′–3′)**	**Amplicon size (bp)**	**References**
*Bartonella* spp.	*gltA*	bart781 bart1137	GGGGACCAGCTCATGGTGG AATGCAAAAAGAACAGTAAACA	380–400	Norman et al., 1995
*Rickettsia* spp.	*gltA*	Rsfg877 Rsfg1258	GGGGGCCTGCTCACGGCGG ATTGCAAAAAGTACAGTGAACA	381	Regnery et al., 1991
*Borrelia* spp.	23S rRNA	Bo_bu_sl_23S_F Bo_bu_sl_23S_R Bo_bu_sl_23S_P	GAGTCTTAAAAGGGCGATTTAGT CTTCAGCCTGGCCATAAATAG AGATGTGGTAGACCCGAAGCCGAGT	73	Michelet et al., 2014
*Borrelia burgdorferi, sensu stricto*	*rpoB*	Bo_bu_rpoB_F Bo_bu_rpoB_R, Bo_bu_rpoB_P	GCTTACTCACAAAAGGCGTCTT GCACATCTCTTACTTCAAATCCT AATGCTCTTGGACCAGGAGGACTTTCA	83	Michelet et al., 2014
*Borrelia garinii*	*rpoB*	Bo_ga_rpoB_F Bo_ga_rpoB_R Bo_ga_rpoB_P	TGGCCGAACTTACCCACAAAA ACATCTCTTACTTCAAATCCTGC TCTATCTCTTGAAAGTCCCCCTGGTCC	88	Michelet et al., 2014
*Borrelia afzelii*	*fla*	Bo_af_fla_F Bo_af_fla_R Bo_af_fla_P	GGAGCAAATCAAGATGAAGCAAT TGAGCACCCTCTTGAACAGG TGCAGCCTGAGCAGCTTGAGCTCC	116	Michelet et al., 2014
*Borrelia valaisiana*	*ospA*	Bo_va_ospA_F Bo_va_ospA_R Bo_va_ospA_P	ACTCACAAATGACAGATGCTGAA GCTTGCTTAAAGTAACAGTACCT TCCGCCTACAAGATTTCCTGGAAGCTT	135	Michelet et al., 2014
*Borrelia lusitaniae*	*rpoB*	Bo_lus_rpoB_F Bo_lus_rpoB_R Bo_lus_rpoB_P	CGAACTTACTCATAAAAGGCGTC TGGACGTCTCTTACTTCAAATCC TTAATGCTCTCGGGCCTGGGGGACT	87	Michelet et al., 2014
*Borrelia spielmanii*	*fla*	Bo_spi_fla_F Bo_spi_fla_R Bo_spi_fla_P	ATCTATTTTCTGGTGAGGGAGC TCCTTCTTGTTGAGCACCTTC TTGAACAGGCGCAGTCTGAGCAGCTT	71	Michelet et al., 2014
*Borrelia bissettii*	*rpoB*	Bo_bi_rpoB_F Bo_bi_rpoB_R Bo_bi_rpoB_P	GCAACCAGTCAGCTTTCACAG CAAATCCTGCCCTATCCCTTG AAAGTCCTCCCGGCCCAAGAGCATTAA	118	Michelet et al., 2014
*Borrelia miyamotoi*	*glpQ*	B_miya_glpQ_F B_miya_glpQ_R B_miya_glpQ_P	CACGACCCAGAAATTGACACA GTGTGAAGTCAGTGGCGTAAT TCGTCCGTTTTCTCTAGCTCGATTGGG	94	Michelet et al., 2014
*Anaplasma phagocytophilum*	*msp2*	An_ph_msp2_F An_ph_msp2_R An_ph_msp2_P	GCTATGGAAGGCAGTGTTGG GTCTTGAAGCGCTCGTAACC AATCTCAAGCTCAACCCTGGCACCAC	77	Michelet et al., 2014
*Candidatus* Neoehrlichia mikurensis	*groEL*	Neo_mik_groEL_F Neo_mik_groEL_R Neo_mik_groEL_P	AGAGACATCATTCGCATTTTGGA TTCCGGTGTACCATAAGGCTT AGATGCTGTTGGATGTACTGCTGGACC	96	Michelet et al., 2014
*TBEV*	*3′ non-coding region*	F-TBE 1 R-TBE 1 TBE-Probe-WT	GGGCGGTTCTTGTTCTCC ACACATCACCTCCTTGTCAGACT TGAGCCACCATCACCCAGACACA	67	Schwaiger and Cassinotti, 2003
	non-structural protein NS5	Outer primers: FSM-1 FSM-2	GAGGCTGAACAACTGCACGA GAACACGTCCATTCCTGATCT	357	Schwaiger and Cassinotti, 2003
	non-structural protein NS5	Inner primers: FSM-li FSM-2i	ACGGAACGTGACAAGGCTAG GCTTGTTACCATCTTTGGAG	251	Schwaiger and Cassinotti, 2003

### Detection of co-infections with other tick-borne pathogens

*Bartonella* spp. and *Rickettsia* spp.: All DNA samples positive for *Borrelia* genospecies were screened for the presence of *Bartonella* spp. and *Rickettsia* spp. via PCR. Detection was carried out using specific primers targeting the *gltA* gene of both *Bartonella* spp. (Norman et al., 1995) and *Rickettsia* spp. (Table 1) (Regnery et al., 1991). PCR products were amplified using the Thermo Scientific Phusion High-Fidelity PCR Kit (Thermo Scientific, USA). Each reaction was carried out in a 20 μl reaction volume containing 5 μl tick DNA, 4 μl 5 X PCR buffer, 200 μM of each dNTP, 0.5 μM of each primer, and 0.4 U phusion DNA polymerase. Thermal conditions were 98°C for 30 s, followed by 35 cycles at 98°C for 10 s, 52°C (for *Bartonella* spp.) or 56°C (for *Rickettsia* spp.) for 30 s, and 72°C for 30 s, with a final elongation at 72°C for 10 min. The 380–400 bp *Bartonella*-specific fragment and the 381 bp *Rickettsia*-specific fragment were sequenced by Eurofins MWG Operon (Ebesberg, Germany). Sequences were assembled using BioEdit software (Hall, 1999), and compared to the NCBI database for species identification.

*A. phagocytophilum*, “*C*. N. mikurensis”: Real-time PCR assays were performed using primers and probes targeting the *msp2* gene for *A. phagocytophilum*, and the *groEL* gene for “*C*. N. mikurensis” (Table 1) (Michelet et al., 2014). All fluorogenic probes were synthesized with a 6-carboxy-fluorescein (FAM) reporter molecule attached to the 5′ end and a Black Hole Quencher 1 (BHQ1) at the 3′ terminus. Real-time Taqman PCR assays were performed in a final volume of 12 μl using the LightCycler® 480 Probe Master mix (Roche Applied Science, Germany) at 1 X final concentration, with primers and probes at 200 nM, and 2 μl DNA. Negative (water) and positive controls were included with each run. Thermal cycling conditions were as follows: 95°C for 5 min, 45 cycles at 95°C for 10 s, then 60°C for 15 s, with a final cooling step at 40°C for 10 s.

TBEV: RNA samples were screened for tick-borne encephalitis virus (TBEV) by rRT-PCR targeting a 3′ non-coding region of the TBEV genome with specific primers and probes (Table 1) (Schwaiger and Cassinotti, 2003). rRT-PCR Taqman assays were performed in a final volume of 20 μl using the LightCycler® 480 RNA Master Hydrolysis Probes master mix (Roche Applied Science, Germany) at 1 X final concentration, with 0.5 μM specific primers and 0.25 μM probes, 3.25 mM manganese acetate [Mn(OAc)_2_] and 2 μl RNA. Positive and negative (water) controls were included in each run. rRT-PCR thermal cycling conditions were as follows: 63°C for 3 min, 95°C for 30 s, 45 cycles at 95°C for 10 s, then 60°C for 30 s, followed by cooling at 40°C for 10 s. Positive samples were used for one-step RT-PCR and nested PCR targeting the non-structural protein gene *NS5* (Puchhammer-Stockl et al., 1995). Positive RNA samples were reverse transcribed using Titan One Tube RT-PCR Systems (Roche Applied Science, Germany) in a 50 μl reaction volume containing 10 μl tick RNA, 10 μl 5 X RT-PCR buffer, 200 μM of each dNTP, 0.4 μM of each primer, 1 μl enzyme mix and 500 μM dithiothreitol (DTT). Thermal cycling conditions were 50°C for 30 min, 95°C for 3 min, followed by 35 cycles at 95°C for 30 s, 40°C for 30 s, and 72°C for 30 s, with a final step at 72°C for 5 min. Nested PCR was performed using the Thermo Scientific Phusion High-Fidelity PCR Kit (Thermo Scientific, USA). PCRs were carried out in a 50 μl reaction volume containing 5 μl tick cDNA, 10 μl 5 X PCR buffer, 200 μM of each dNTP, 0.5 μM of each internal primer, and 1 U Phusion DNA polymerase. Thermal conditions were 98°C for 30 s, followed by 35 cycles at 98°C for 10 s, 53 C for 30 s, and 72°C for 60 s, and a final elongation at 72°C for 10 min.

### Statistical analysis

The data were processed using IBM SPSS® Statistics version 21 (IBM® Corporation, Chicago, IL, USA) software. One-way analysis of variance (ANOVA) tests (Bonferroni *post-hoc* test) were performed on tick infection rates for geographic groups. Prevalence rates were compared between tick developmental stages using independent sample *T*-tests. In cases of statistical significance, *p*-values are presented in parentheses. Differences were considered statistically significant when *p* < 0.05.

## Results

### Tick collection

Using the dragging protocol, we collected 557 ticks belonging to three different species: *I*. *ricinus* (from all sampling areas), *Dermacentor reticulatus* (only from sampling area 2), and *H. punctata* (from sampling areas 2 and 3). *I. ricinus* ticks represented 95.9% (534/557) of the collected ticks, with 77 adult ticks and 457 nymphs, *D. reticulatus* represented 0.7% (4/557) of collected ticks with four adults, and *H. punctata* represented 3.4% (19/557) of collected ticks with three adults and 16 nymphs.

### *Borrelia* genospecies detected in ticks

*Borrelia* spp. DNA was identified in 138/534 (25.8%) of *I. ricinus* ticks collected from all sampling sites, in none of the four *D. reticulatus* ticks, and in 1/19 (5.3%) *H. punctata* collected from area 2. *Borrelia* genospecies found in *H. punctata* was identified as *B. miyamotoi*. The *B. miyamotoi*-positive *H. punctata* was not co-infected with other screened tick-borne pathogens.

In *I. ricinus* ticks, *Borrelia* spp. infection rates were not significantly different between adults (26.0%; 20/77) and nymphs (25.8%; 118/457) (*p* > 0.05). *Borrelia* spp. prevalence was significantly higher in *I. ricinus* ticks collected from area 3 in the southernmost sector of eastern Romania, than in ticks from areas 1 (*p* < 0.05) and 2 (*p* < 0.05) (40.0% vs. 25.0% or 25.0% in adults, and 40.0% vs. 22.0% or 16.7% in nymphs).

Eight genospecies of *Borrelia* were identified: *B*. *garinii, B. afzelii, B. valaisiana, B. lusitaniae, B. spielmanii, B. burgdorferi* s.s., *B. bissettii*, and *B. miyamotoi*. The mean infection rate was 14.8% for *B. garinii* (16.9% of the adults and 14.4% of the nymphs), 8.8% for *B. afzelii* (7.8% of adults and 9.0% of nymphs), 5.1% for *B. valaisiana* (5.2% of adults and 5.0% of nymphs), 4.9% for *B. lusitaniae* (5.2% of adults and 4.8% of nymphs), 1.1% for *B. spielmanii* (1.3% of adults and 1.1% of nymphs), 0.9% for *B. miyamotoi* (1.3% of adults and 0.9% of nymphs), 0.4% for *B. burgdorferi* s.s. (0.4% of nymphs), and 0.2% for *B. bissettii* (0.2% of nymphs).

The infection rates of different *Borrelia* genospecies in *I. ricinus* ticks according to collection site is shown in Table 2. Ticks collected from area 1 were more frequently infected with *B. afzelii* but did not register statistical significance. Ticks from area 3 were more frequently infected with *B*. *garinii*, with a statistically significant difference only when compared to *B. garinii* infection rates in area 2 (*p* < 0.05). *B. valaisiana* also demonstrated a high infection rate in area 3, which was statistically different to the prevalence rate obtained in area 2 (*p* < 0.05). *B. lusitaniae* prevalence was significantly higher in area 3 than in areas 1 and 2 (*p* < 0.05). *B. spielmanii* was detected in areas 2 and 3 with similar infection rates; *B. burgdorferi* s.s. was only detected in areas 1 and 2, and *B. bissettii* was only detected in area 1. Relapsing fever agent *B. miyamotoi* was only detected in areas 1 and 2 (see Table 2).

**Table 2:** ***Borrelia* genospecies identified in *Ixodes ricinus* ticks (%)**.

**Sampling area**	**Tick samples (no. of samples)**	***B. garinii***	***B. afzelii***	***B. valaisiana***	***B. lusitaniae***	***B. spielmanii***	***B. burgdorferi* s.s**.	***B. bissettii***	***B. miyamotoi***
Sampling area 1	Adults (8)	2/25.0	1/12.5		1/12.5				
	Nymphs (59)	7/11.9	8/13.6				1/1.7	1/1.7	1/1.7
	Total (67)	9/13.4	9/13.4		1/1.5		1/1.5	1/1.5	1/1.5
Sampling area 2	Adults (64)	9/14.1	4/6.3	4/6.3	2/3.1	1/1.6			1/1.6
	Nymphs (233)	19/8.2	18/7.7	6/2.6	4/1.7	2/0.9	1/0.4		3/1.3
	Total (297)	28/9.4	22/7.4	10/3.4	6/2.0	3/1.0	1/0.3		4/1.4
Sampling area 3	Adults (5)	2/40.0	1/20.0		1/20.0				
	Nymphs (165)	40/24.2	15/9.1	17/10.3	18/10.9	3/1.8			
	Total (170)	42/24.7	16/9.4	17/10.0	19/11.2	3/1.8			
Total	Adults (77)	13/16.9	6/7.8	4/5.2	4/5.2	1/1.3			1/1.3
	Nymphs (457)	66/14.4	41/9.0	23/5.0	22/4.8	5/1.1	2/0.4	1/0.2	4/0.9
	Total (534)	79/14.8	47/8.8	27/5.1	26/4.9	6/1.1	2/0.4	1/0.2	5/0.9

### Co-infections in *Ixodes ricinus*

Co-infections were identified in 16.7% *I. ricinus* ticks (89/534), which accounted for 64.5% (89/138) of all infected ticks. Co-infection prevalence was 14.3% (11/77) in adults and 17.1% (78/457) in nymphs. Co-infection rates were significantly higher in area 3 (27.7%; 47/170) than in areas 1 and 2 (14.9%; 10/67 and 10.8%; 32/297, respectively) resulting in a statistically significant difference only when compared to area 2 (*p* < 0.05).

### Co-infection between *Borrelia* genospecies

The overall prevalence of *B. burgdorferi* s.l. genospecies co-infection was 9.7% (52/534; 9.1% of adults and 9.8% of nymphs). Forty-five *I. ricinus* ticks were co-infected with two *B. burgdorferi* s.l. genospecies. The most frequent *Borrelia* genospecies association was between *B. garinii* and *B. afzelii* (23/534; 4.3%), followed by *B. garinii* and *B. lusitaniae* (16/534; 3.0%). Triple *B. burgdorferi* s.l. genospecies co-infection was identified in seven *I. ricinus* ticks, four of which were infected with *B. garinii, B. afzelii*, and *B. valaisiana* (Table 3). Co-infection rates between *Borrelia* genospecies were significantly higher in area 3 [15.3% (26/170; 40.0% adults and 14.5% nymphs)] than in areas 1 and 2 [8.9% (6/67; 10.1% nymphs) and 6.7% (20/297; 7.8% adults and 6.4% nymphs)], respectively (*p* < 0.05).

**Table 3:** **Co-infection between *Borrelia* genospecies and other tick-borne pathogens**.

**Pathogen associations**	**Area 1**			**Area 2**			**Area 3**			**Total No**.		
	**A**	**N**	**Total**	**A**	**N**	**Total**	**A**	**N**	**Total**	**A**	**N**	**Total**
*B. garinii + B. afzelii*		5	5	3	9	12	1	5	6	4	19	23
*B. garinii + B. lusitaniae*					1	1	1	14	15	1	15	16
*B. garinii + B. spielmanii*					2	2		1	1		3	3
*B. afzelii + B. bissettii*		1	1								1	1
*B. afzelii + B. lusitaniae*					1	1					1	1
*B. garinii + B. valaisiana*								1	1		1	1
*B. garinii + B. afzelii + B. valaisiana*					1	1		3	3		4	4
*B. garinii + B. afzelii + B. lusitaniae*					1	1					1	1
*B. garinii + B. afzelii + B. spielmanii*				1		1				1		1
*B. garinii + B. valaisiana + B. lusitaniae*				1		1				1		1
*B. garinii* + *R. monacensis*					1	1		1	1		2	2
*B. valaisiana* + *Bartonella* spp.				1	1	2				1	1	2
*B.afzelii* + “*C* N mikurensis”					2	2					2	2
*B. valaisiana* + “*C* N mikurensis”								2	2		2	2
*B. valaisiana + R. monacensis*				1		1				1		1
*B. valaisiana* + *Rickettsia* spp.					1	1					1	1
*B. afzelii + Rickettsia* spp.		1	1								1	1
*B. miyamotoi* + “*C* N mikurensis ”		1	1								1	1
*B. miyamotoi* + *Bartonella* spp.					1	1					1	1
*B. garinii + Bartonella* spp.					1	1					1	1
*B. spielmanii + A. phagocytophilum*								1	1		1	1
*B. garinii + Rickettsia* spp.								1	1		1	1
*B. garinii + “C* N mikurensis”								1	1		1	1
*B. afzelii + R. helvetica*								1	1		1	1
*Borrelia* spp. + “*C* N mikurensis”								1	1		1	1
*Borrelia* spp. + *Bartonella* spp.					1	1					1	1
*B. garinii + B*. *afzelii + Rickettsia* spp.	1		1		1	1		1	1	1	2	3
*B. garinii + B*. *lusitaniae + Rickettsia* spp.	1		1					1	1	1	1	2
*B. garinii + B. afzelii + R. monacensis*								2	2		2	2
*B. valaisiana + B. spielmanii + R. monacensis*								1	1		1	1
*B. garinii + B. valaisiana + R. helvetica*								1	1		1	1
*B. garinii + B. afzelii + A. phagocytophilum*								1	1		1	1
*B. garinii + B. afzelii + “C* N mikurensis”								1	1		1	1
*B. garinii + B. valaisiana + “C* N mikurensis”								1	1		1	1
*B. garinii* + *R. helvetica* + *Bartonella* spp.								1	1		1	1
*B. valaisiana + R. monacensis* + “*C* N mikurensis”								1	1		1	1
*B. valaisiana + Rickettsia* spp. + “*C* N mikurensis”								1	1		1	1
*Borrelia* spp. + *R. monacensis* + “*C* N mikurensis”								1	1		1	1
*B. garinii* + *B. afzelii* + *B. lusitaniae* + “*C* N mikurensis”					1	1					1	1
Total	2	8	10	7	25	32	2	45	47	11	78	89

*A (Adult ticks), N (Nymphs), “Candidatus Neoehrlichia mikurensis” (“C. N. mikurensis”)*.

### Co-infection between *Borrelia* genospecies and other tick-borne pathogens

Co-infections of *Borrelia* genospecies with other tick-borne pathogens occurred in 6.9% of all collected *I. ricinus* ticks (37/534; 5.2% of adults and 7.2% of nymphs), and were observed in ticks from all three sampling areas as follows: 6.0% of ticks from area 1 (4/67; 25.0% of adults and 3.4% of nymphs), 4.0% of ticks from area 2 (12/297; 3.1% of adults and 4.3% of nymphs), and 12.4% of ticks from area 3 (21/170; 12.7% of nymphs).

Dual co-infection between one *Borrelia* genospecies and another pathogen occurred in 20 *I. ricinus* ticks (20/534; 3.7%). The most frequent co-infection was between *Borrelia* spp. and *Rickettsia* spp., and between *Borrelia* spp. and “*C*. N. mikurensis”, where each association was detected in seven different *I. ricinus* ticks. Five samples tested positive for *Borrelia* spp. and *Bartonella* spp., and one tick was co-infected with *Borrelia* spp. and *A. phagocytophilum*.

Association of two *Borrelia* genospecies with another pathogen genus was detected in 12 ticks (12/534; 2.4%), of which nine ticks tested positive for co-infection with two *Borrelia* genospecies and one *Rickettsia* spp. We detected two samples infected with *Borrelia* genospecies and “*C*. N. mikurensis”, and one sample co-infected with two *Borrelia* genospecies and *A. phagocytophilum*.

We also recorded four (4/534; 0.7%) ticks infected with one *Borrelia* genospecies and two other pathogen genera: Three ticks positive for the association of *Borrelia* spp. + *Rickettsia* spp. + “*C*. N. mikurensis”, and one tick positive for *Borrelia* spp., *Rickettsia* spp., and *Bartonella* spp. One tick was positive for three different *Borrelia* genospecies (*B. garinii, B. afzelii, B. lusitaniae*), and “*C*. N. mikurensis” (Table 3).

We did not identify co-infections between *Borrelia* genospecies with tick-borne encephalitis virus.

## Discussion

Using a powerful high-throughput tool we performed a comprehensive overview of the epidemiological status of Lyme spirochetes circulating and co-circulating with other important tick-borne pathogens in eastern Romanian ticks. The most important findings were: (1) the identification of seven different genospecies of the Lyme spirochete group—including *B. miyamotoi*—with an overall high *Borrelia* prevalence rate (25.8% of ticks infected); (2) the frequency of co-infection: Among *Borrelia*-infected ticks, 64.5% were co-infected; (3) the unexpected high infection and co-infection rates of ticks collected from the Danube Delta biosphere (area 3). Together, these results may have important implications in terms of public health issues in a country where Lyme disease surveillance has only been implemented since 2009.

In our study, the overall *Borrelia* prevalence rate across all sampling areas (25.8%), was much higher than compared to previous studies of this Lyme disease agent in questing *I. ricinus* ticks from Romania that reported prevalences ranging from 3.8 to 18% (Coipan and Vladimirescu, 2011; Kalmar et al., 2013). This result is likely due to high sensitivity of our techniques which combines pathogen DNA pre-amplification steps with specific quantitative amplification of target pathogen DNA.

### Different *Borrelia* genospecies identified

*B. garinii* and *B. afzelii* were the most abundant *Borrelia* genospecies in our study, as has been previously reported in Romanian ticks (Coipan and Vladimirescu, 2010, 2011; Kalmar et al., 2013; Briciu et al., 2014). Several other studies have shown that certain *Borrelia* genospecies have preferred reservoir hosts; *B. garinii* is mostly associated with birds, whereas *B. afzelii* genospecies are predominantly isolated from medium-sized and small mammals, especially rodents (Dubska et al., 2009; Rizzoli et al., 2011; Mannelli et al., 2012). Hence *Borrelia* genospecies prevalence in specific areas is thus closely related to the abundance of competent reservoir hosts (Rizzoli et al., 2011). The high prevalence of *B. garinii* might be due to the increased density of bird-based enzootic cycles in eastern Romania, especially in area 3 (Danube Delta region), which represents an important stopover site for over 300 different avian species migrating between northern Eurasia and Africa (Sándor et al., 2014). Northward, near Iaşi, 129 bird species have been identified (Gache, 2016) including song thrushes (*Turdus philomelos*) and blackbirds (*T. merula*), reported to be the principle *B. garinii* reservoir in central Europe (Taragel'ova et al., 2008). *B. afzelii* was the second most prevalent genospecies, and is predominantly cycled throughout Europe via rodent species (*Apodemus sylvaticus, A. agrarius, A. flavicollis*, and *Myodes glareolus*), known common tick hosts (Mihalca et al., 2012b). The 32 species of wild rodents found in Romania (Mihalca et al., 2012b) emphasize the significant biodiversity and availability of putative *B. afzelii* reservoir hosts.

*Borrelia valaisiana* and *B. lusitaniae* had similar infection rates in questing ticks, and *B. lusitaniae* was detected at all three sampling areas. The pathogenicity of these genospecies is still uncertain, even though there is evidence of their presence in skin biopsies and cerebrospinal fluid of patients with Lyme disease (Diza et al., 2004; Rauter and Hartung, 2005). Similarly to *B. garinii, B. valaisiana* is more commonly identified in birds, while *B. lusitaniae* is mainly associated with lizards (Rizzoli et al., 2011; Mannelli et al., 2012). We report higher *B. valaisiana* and *B. lusitaniae* infection rates in Romania compared to previous studies (de la Fuente et al., 2008; Kalmar et al., 2013), which may be a result of high reservoir host density. In Romania, lizards are commonly found, the green lizard (*Lacerta viridis*) and sand lizard (*Lacerta agilis*) are both widely distributed throughout our study areas (Cogalniceanu et al., 2013). The role of these host species in the transmission cycle of *B. lusitaniae* has been studied in Slovakia, Poland, and Romania (Majlathova et al., 2006; Majláthová et al., 2008), thus it is likely that their presence induces favorable conditions for *B. lusitaniae* persistence in eastern Romania.

We also detected three other *B. burgdorferi* s.l. group genospecies at low levels: *B*. *burgdorferi* s.s., *B. spielmanii*, and *B. bissettii*. *B. burgdorferi* s.s. and *B. spielmanii* are recognized human pathogens in Europe, while *B. bissettii*'s role in human disease remains unclear (Briciu et al., 2014). Small mammals and birds are the preferred reservoir hosts for *B. burgdorferi* s.s., dormice for *B. spielmanii*, and rodents for *B. bissettii* (Eisen et al., 2009).

*Borrelia miyamotoi* represents the only known relapsing fever group agent transmitted by *Ixodes* species (Potkonjak et al., 2016) with probable reservoir hosts of field mice, birds, and voles (Krause et al., 2015). *B. miyamotoi* has sporadic geographic distribution and has been detected in many European countries, in North America, and Asia (Crowder et al., 2014). In 2016, Kalmár et al. first reported the identification of this agent in questing ticks from central Romania (Kalmár et al., 2016). Our study confirms the existence of this pathogen in ixodid ticks from eastern Romania at a low infection rate and with limited geographic expansion.

### Prevalence according to collection sites

*Borrelia* genospecies were detected at all studied areas. Infection rates in ticks from area 1 (deciduous and mixed forest vegetation) was similar to area 2 (urban area, *B. burgdorferi* s.l. was detected in 18.5% of ticks), thus highlighting a significant Lyme disease risk in area 2 a heavily frequented recreational hotspot. Lyme spirochetes prevalence was surprisingly higher in ticks collected from the forested but arid area 3 in southeastern Romania (40.0%) than in ticks from the other two more northern areas, which both represent a more typical landscape for *I. ricinus* and *Borrelia* spirochetes.

Sampling area 3 was located in the Danube Delta Biosphere Reserve known to harbor a wide variety of mammals, migratory and resident bird species all previously described as tick hosts (Sándor et al., 2014). Thus, the combination of abundant bird fauna and the diversity of tick species might explain the high prevalence of bird-associated *B. garinii* and *B. valaisiana*.

Interestingly, the second most prevalent genospecies in questing ticks from area 3 was *B. lusitaniae* (11.2%), which was also more prevalent in area 3 than the other two studied areas. Under natural conditions *Borrelia lusitaniae* circulates between lizards and ticks (Majláthová et al., 2008), and three lizard species (*Podarcis muralis, Lacerta viridis, L. agilis*) are designated as *B. lusitaniae* reservoirs (Majláthová et al., 2008). In area 3, many lizard species have been identified (*L. agilis, L. viridis, L. trilineata, P. muralis, P. tauricus*) (Cogalniceanu et al., 2013), and likely contribute to the high *B. lusitaniae* tick infection rate. Under conditions of relatively low humidity, such as in area 3, nymphs seek out their hosts from low growing vegetation, which promotes attachment to small animals during the warmer parts of the day. During these warmer daytime periods, lizards are active and mice become inactive, preferentially favoring tick attachment to lizards and subsequently, *B. lusitaniae* dominance (Mannelli et al., 2012).

The rodent-associated *Borrelia* genospecies, *Borrelia afzelii*, was detected in ticks from all three geographical areas with a relatively high infection rate, which could be due to the high diversity and abundance of rodent species in Romania (Mihalca et al., 2012b). In fact, studies focusing on *B. burgdorferi* genospecies in Romanian ticks, reported *B. afzelii* to be the most frequent (Coipan and Vladimirescu, 2010, 2011; Dumitrache et al., 2013; Kalmar et al., 2013; Briciu et al., 2014).

*Borrelia spielmanii* was only detected in areas 2 and 3, at low infection levels. This genospecies is a pathogenic spirochete transmitted by *I. ricinus*, and is associated with garden and hazel dormice (Richter et al., 2011). Dormice have a low population density in Romania (Drăgoi and Faur, 2013), which could explain the low prevalence of *B. spielmanii* in our study.

*B. miyamotoi* was only identified at areas 1 and 2 in our study, and at a low infection rate (1.5% in area 1, and 1.3% in area 2). No human cases of *B. miyamotoi* infection have been identified in Romania thus far.

### Prevalence in adult ticks and nymph

Usually, adult *I. ricinus* are more frequently infected with *Borrelia* spp. than nymphs, and as host-seeking adult ticks require two blood meals during their development, compared to one for nymphs, the likelihood of retrieving the pathogen from infected hosts is increased (Schouls et al., 1999; Geller et al., 2013; Kalmar et al., 2013; Soleng and Kjelland, 2013). Our observed adult and nymph infection rates did not follow this pattern, as *I. ricinus* adults were only slightly more frequently infected with *Borrelia* spp. than nymphs. This could be attributed to the high abundance of *I. ricinus* nymphs in study areas, which is known to positively correlate with a high rate of infected nymphs (Vennestrom et al., 2008). Additionally, adults feed on host species that are not *B. burgdorferi* s.l.-transmission competent, such as deer, resulting in a similar prevalence in adults compared to nymphs (Gray et al., 1999). Larval stages are more frequently infected with *Borrelia* genospecies as a result of feeding mostly on rodents, birds, and lizards, and thus acquiring the infection after a blood meal from an infected host, or by co-feeding in the vicinity of an infected nymph (van Duijvendijk et al., 2016).

### Tick co-infection and impact on health

In this study, we identified several pathogens co-existing within ticks. Co-infections were detected in 14.3% of adult ticks and in 17.1% of nymphs. Moreover, associations between *Borrelia* genospecies and between *Borrelia* and other tick-borne pathogens were detected in 64.5% of infected ticks. Recently, tick co-infections with different tick-borne pathogens have been more frequently reported (Reis et al., 2011; Lommano et al., 2012; Eshoo et al., 2014; Moutailler et al., 2016). This is particularly the case for *I. ricinus*, due to its capacity to feed on a broad variety of vertebrate species that can host multiple tick-borne pathogens (Reis et al., 2011). In our study, multiple *Borrelia* genospecies co-existence was identified in 9.7% of ticks, representing 37.7% of *Borrelia*-positive *I. ricinus*. Multiple *Borrelia* infections in individual ticks may occur via several mechanisms, such as: Superinfection of ticks with prior transovarial infection, infection with multiple pathogens via co-feeding, multiple infection after feeding on hosts positive for several *Borrelia* genospecies, and successive infectious blood meals (Rauter and Hartung, 2005).

In our study, the most common co-infection in individual ticks was between *B. afzelii* and *B. garinii* (4.3%), as previously shown (Moutailler et al., 2016). As *B. afzelii* and *B. garinii* have different reservoir hosts (rodents and birds respectively) (Mannelli et al., 2012), infection of individual ticks with both genospecies could be the outcome of several possibilities: Immature ticks feeding on both reservoir hosts (as it is the case for positive adult ticks), infection of larvae by co-feeding (Moutailler et al., 2016), or co-infection of reservoir hosts with both *B. afzelii* and *B. garinii*.

The second most frequent *Borrelia* genospecies association was between *B. garinii* and *B. lusitaniae*. This mixed infection was predominantly detected in area 3 (Table 3) with many bird varieties existing as common reservoir hosts for both genospecies (Cogalniceanu et al., 2013; Sándor et al., 2014).

We detected 6.9% of ticks positive for the following co-infection associations: (i) dual co-infection with one *Borrelia* genospecies and another pathogen (20 ticks); (ii) co-infection of two *Borrelia* genospecies with another pathogen genus (12 ticks); (iii) association of a *Borrelia* genospecies with two other tick-borne pathogens (4 ticks); and (iv) co-infection with three *Borrelia* genospecies and one different pathogen genus (1 tick).

The most common tick co-infection was between *Borrelia* genospecies and *Rickettsia monacensis*, followed by the association of different *Borrelia* genospecies and “*C*. N. mikurensis”. Birds (especially blackbirds—*Turdus merula*) are reported as competent reservoir hosts for *R. monacensis* (Marcutan et al., 2016), a role shared with ticks. The association of “*C*. N. mikurensis” with *B. garinii* and *B. valaisiana* is likely due to shared reservoirs, while *R. monacensis* and *B. afzelii* co-infection could be the result of co-feeding.

Wild rodents are reservoir hosts for several *Borrelia* genospecies as well as “*C*. N. mikurensis” which appears to follow the same distribution pattern as the Lyme disease spirochetes (Burri et al., 2014). As “*C*. N. mikurensis” transovarial transmission cannot occur, ticks are likely to have been infected with both pathogens from the same host during feeding (Andersson et al., 2013). This type of multiple infection was most frequent in area 3, again likely due to reservoir host abundance. We also detected a small number of associations between *Borrelia* with *A. phagocytophilum* and *Bartonella* spp., adding further evidence to the hypothesis that ticks carry a large number of tick-borne pathogens in eastern Romania.

*I. ricinus* nymphs represent the most high-risk stage to public health due to their small size, their marked anthropophily, their abundance, and capacity to remain attached to hosts for long durations (Vassallo et al., 2000). In addition to their crucial role as vectors of pathogens, we identified high levels of co-infection which might have important implications for human co-infection. Indeed, the multiple pathogens present in individual ticks could be co-transmitted, either resulting in a co-infection which enhances disease severity—as has been demonstrated for concurrent babesiosis and Lyme disease (Moutailler et al., 2016)—or which might evolve with atypical symptoms, resulting in diagnostic difficulties.

Our findings confirm that ticks are not only important pathogen vectors in forested areas, but also in urban situations. Worth highlighting is the risk that Lyme disease poses in areas with increased recreational activity, as we found high *Borrelia* genospecies infection rates in questing *I. ricinus* ticks collected from urban sites. As such, these areas require improved surveillance. We have also identified new risk areas, and have generated detailed data on the occurrence of novel and known zoonotic pathogens, which will act as a solid foundation for further studies examining the risk of human tick-borne disease.

## Author contributions

CR collected the samples, performed the experiment, and wrote the MS. IP and DP collected the samples. SM designed the studies. AM and GS discussed the results and wrote the MS. MT designed the studied, analyzed the results and wrote the MS.

### Conflict of interest statement

The authors declare that the research was conducted in the absence of any commercial or financial relationships that could be construed as a potential conflict of interest.

## References

[B1] Agoulon A. Malandrin L. Lepigeon F. Venisse M. Bonnet S. Becker C. A.. (2012). A vegetation index qualifying pasture edges is related to *Ixodes ricinus* density and to *Babesia divergens* seroprevalence in dairy cattle herds. Vet. Parasitol. 185, 101–109. 10.1016/j.vetpar.2011.10.022 22079425

[B2] Andersson M. Bartkova S. Lindestad O. Raberg L. (2013). Co-infection with 'Candidatus Neoehrlichia Mikurensis' and *Borrelia afzelii* in *Ixodes ricinus* ticks in southern Sweden. Vector Borne Zoonot. Dis. 13, 438–442. 10.1089/vbz.2012.1118 23590321

[B3] Briciu V. T. Meyer F. Sebah D. Tatulescu D. F. Coroiu G. Lupse M.. (2014). Real-time PCR-based identification of *Borrelia burgdorferi* sensu lato species in ticks collected from humans in Romania. Ticks Tick Borne Dis. 5, 575–581. 10.1016/j.ttbdis.2014.04.007 24986749

[B4] Burri C. Schumann O. Schumann C. Gern L. (2014). Are Apodemus spp. mice and myodes glareolus reservoirs for *Borrelia miyamotoi*, Candidatus Neoehrlichia mikurensis, *Rickettsia helvetica, R. monacensis* and *Anaplasma phagocytophilum*? Ticks Tick Borne Dis. 5, 245–251. 10.1016/j.ttbdis.2013.11.007 24582511

[B5] Chowdri H. R. Gugliotta J. L. Berardi V. P. Goethert H. K. Molloy P. J. Sterling S. L.. (2013). *Borrelia miyamotoi* infection presenting as human granulocytic anaplasmosis: a case report. Ann. Intern. Med. 159, 21–27. 10.7326/0003-4819-159-1-201307020-00005 23817701

[B6] Clark K. L. Leydet B. F. Threlkeld C. (2014). Geographical and genospecies distribution of *Borrelia burgdorferi* sensu lato DNA detected in humans in the USA. J. Med. Microbiol. 63, 674–684. 10.1099/jmm.0.073122-0 24568883

[B7] Cogalniceanu D. Rozylowicz L. Szekely P. Samoila C. Stanescu F. Tudor M. (2013). Diversity and distribution of reptiles in Romania. Zookeys 341, 49–76. 10.3897/zookeys.341.5502 PMC3800809 24146598

[B8] Coipan E. C. Vladimirescu A. F. (2010). First report of lyme disease spirochetes in ticks from Romania (Sibiu County). Exp. Appl. Acarol. 52, 193–197. 10.1007/s10493-010-9353-0 20232115

[B9] Coipan E. C. Vladimirescu A. F. (2011). *Ixodes ricinus* ticks (Acari: Ixodidae): vectors for Lyme disease spirochetes in Romania. Exp. Appl. Acarol. 54, 293–300. 10.1007/s10493-011-9438-4 21431930

[B10] Cosson J. F. Michelet L. Chotte J. Le Naour E. Cote M. Devillers E.. (2014). Genetic characterization of the human relapsing fever *spirochete Borrelia miyamotoi* in vectors and animal reservoirs of Lyme disease spirochetes in France. Parasit. Vectors 7:233. 10.1186/1756-3305-7-233 24886071 PMC4039308

[B11] Crowder C. D. Carolan H. E. Rounds M. A. Honig V. Mothes B. Haag H.. (2014). Prevalence of *Borrelia miyamotoi* in Ixodes ticks in Europe and the United States. Emerg. Infect. Dis. 20, 1678–1682. 10.3201/eid2010.131583 25280366 PMC4193165

[B12] de la Fuente J. Estrada-Pena A. Venzal J. M. Kocan K. M. Sonenshine D. E. (2008). Overview: ticks as vectors of pathogens that cause disease in humans and animals. Front. Biosci. 13, 6938–6946. 10.2741/3200 18508706

[B13] Diuk-Wasser M. A. Vannier E. Krause P. J. (2016). Coinfection by ixodes tick-borne pathogens: ecological, epidemiological, and clinical consequences. Trends Parasitol. 32, 30–42. 10.1016/j.pt.2015.09.008 26613664 PMC4713283

[B14] Diza E. Papa A. Vezyri E. Tsounis S. Milonas I. Antoniadis A. (2004). *Borrelia valaisiana* in cerebrospinal fluid. Emerg. Infect. Dis. 10, 1692–1693 10.3201/eid1009.030439 15503409 PMC3320289

[B15] Drăgoi C.-I. Faur M. (2013). Monitoring dormice (Gliridae) populations as a method of evaluating the efficiency of biodiversity management tools in Grădiştea Muncelului – Cioclovina Nature Park, in 5th Symposium for Research in Protected Areas. Mittersill, 143–146.

[B16] Dubska L. Literak I. Kocianova E. Taragelova V. Sychra O. (2009). Differential role of passerine birds in distribution of Borrelia spirochetes, based on data from ticks collected from birds during the postbreeding migration period in Central Europe. Appl. Environ. Microbiol. 75, 596–602. 10.1128/AEM.01674-08 19060160 PMC2632145

[B17] Dumitrache M. O. Matei I. A. Ionica A. M. Kalmar Z. D'Amico G. Siko-Barabasi S.. (2015). Molecular detection of *Anaplasma phagocytophilum* and *Borrelia burgdorferi* sensu lato genospecies in red foxes (*Vulpes vulpes*) from Romania. Parasit. Vectors 8:514. 10.1186/s13071-015-1130-9 26449360 PMC4599586

[B18] Dumitrache M. O. Pastiu A. I. Kalmar Z. Mircean V. Sandor A. D. Gherman C. M.. (2013). Northern white-breasted hedgehogs *Erinaceus roumanicus* as hosts for ticks infected with *Borrelia burgdorferi* sensu lato and *Anaplasma phagocytophilum* in Romania. Ticks Tick Borne Dis. 4, 214–217. 10.1016/j.ttbdis.2012.11.010 23339970

[B19] Egyed L. Elo P. Sreter-Lancz Z. Szell Z. Balogh Z. Sreter T. (2012). Seasonal activity and tick-borne pathogen infection rates of *Ixodes ricinus* ticks in Hungary. Ticks Tick Borne Dis. 3, 90–94. 10.1016/j.ttbdis.2012.01.002 22445929

[B20] Eisen L. Eisen R. J. Mun J. Salkeld D. J. Lane R. S. (2009). Transmission cycles of *Borrelia burgdorferi* and *B. bissettii* in relation to habitat type in northwestern California. J. Vector Ecol. 34, 81–91. 10.1111/j.1948-7134.2009.00010.x 20514140 PMC2876337

[B21] Eshoo M. W. Crowder C. D. Carolan H. E. Rounds M. A. Ecker D. J. Haag H.. (2014). Broad-range survey of tick-borne pathogens in Southern Germany reveals a high prevalence of Babesia microti and a diversity of other tick-borne pathogens. Vector Borne Zoonotic Dis. 14, 584–591. 10.1089/vbz.2013.1498 25072989 PMC4117270

[B22] Gache C. (2016). Bird fauna diversity and habitat evolution in jijioara river valley (romania). travaux du muséum national d'histoire naturelle ≪grigore antipa≫. 582, 73–81. 10.1515/travmu-2016-0008

[B23] Geller J. Nazarova L. Katargina O. Golovljova I. (2013). *Borrelia burgdorferi* sensu lato prevalence in tick populations in Estonia. Parasit. Vectors 6:202. 10.1186/1756-3305-6-202 23837798 PMC3716901

[B24] Gherman C. M. Sandor A. D. Kalmar Z. Marinov M. Mihalca A. D. (2012). First report of *Borrelia burgdorferi* sensu lato in two threatened carnivores: the marbled polecat, *Vormela peregusna* and the European mink, *Mustela lutreola* (Mammalia: Mustelidae). BMC Vet. Res. 8:137. 10.1186/1746-6148-8-137 22901862 PMC3514366

[B25] Golightly L. M. Hirschhorn L. R. Weller P. F. (1989). Fever and headache in a splenectomized woman. Rev. Infect. Dis. 11, 629–637. 10.1093/clinids/11.4.629 2772469

[B26] Gray J. S. Kirstein F. Robertson J. N. Stein J. Kahl O. (1999). *Borrelia burgdorferi* sensu lato in *Ixodes ricinus* ticks and rodents in a recreational park in south-western Ireland. Exp. Appl. Acarol. 23, 717–729. 10.1023/A:1006233700194 10581711

[B27] Grunwaldt E. Barbour A. G. Benach J. L. (1983). Simultaneous occurrence of babesiosis and Lyme disease. N. Engl. J. Med. 308, 1166. 10.1056/NEJM198305123081918 6682178

[B28] Gugliotta J. L. Goethert H. K. Berardi V. P. Telford S. R. III. (2013). Meningoencephalitis from *Borrelia miyamotoi* in an immunocompromised patient. N. Engl. J. Med. 368, 240–245. 10.1056/NEJMoa1209039 23323900 PMC4018741

[B29] Hall T. A. (1999). BioEdit: a user-friendly biological sequence alignment editor and analysis program for Windows 95/98/NT. Nucleic Acids Symp. Ser. 41, 95–98.

[B30] Horowitz H. W. Aguero-Rosenfeld M. E. Holmgren D. McKenna D. Schwartz I. Cox M. E.. (2013). Lyme disease and human granulocytic anaplasmosis coinfection: impact of case definition on coinfection rates and illness severity. Clin. Infect. Dis. 56, 93–99. 10.1093/cid/cis852 23042964

[B31] Hovius J. W. de Wever B. Sohne M. Brouwer M. C. Coumou J. Wagemakers A.. (2013). A case of meningoencephalitis by the relapsing fever spirochaete *Borrelia miyamotoi* in Europe. Lancet 382:658. 10.1016/S0140-6736(13)61644-X 23953389 PMC3987849

[B32] Ionita M. Mitrea I. L. Pfister K. Hamel D. Silaghi C. (2013). Molecular evidence for bacterial and protozoan pathogens in hard ticks from Romania. Vet. Parasitol. 196, 71–76. 10.1016/j.vetpar.2013.01.016 23428204

[B33] Ionita M. Silaghi C. Mitrea I. L. Edouard S. Parola P. Pfister K. (2016). Molecular detection of *Rickettsia conorii* and other zoonotic spotted fever group rickettsiae in ticks, Romania. Ticks Tick Borne Dis. 7, 150–153. 10.1016/j.ttbdis.2015.10.006 26507182

[B34] Kalmar Z. Mihalca A. D. Dumitrache M. O. Gherman C. M. Magdaş C. Mircean V.. (2013). Geographical distribution and prevalence of *Borrelia burgdorferi* genospecies in questing *Ixodes ricinus* from Romania: a countrywide study. Ticks Tick Borne Dis. 4, 403–408. 10.1016/j.ttbdis.2013.04.007 23890805

[B35] Kalmár Z. Sprong H. Mihalca A. Gherman C. Dumitrache M. Coipan E.. (2016). *Borrelia miyamotoi* and candidatus *Neoehrlichia mikurensis* in *Ixodes ricinus* ticks, Romania [letter]. Emerg. Infect. Dis. 22, 550–551. 10.3201/eid2203.150140 26889789 PMC4766875

[B36] Knapp K. L. Rice N. A. (2015). Human coinfection with *Borrelia burgdorferi* and Babesia microti in the United States. J. Parasitol. Res. 2015:587131. 10.1155/2015/587131 26697208 PMC4677215

[B37] Krause P. J. Fish D. Narasimhan S. Barbour A. G. (2015). *Borrelia miyamotoi* infection in nature and in humans. Clin. Microbiol. Infect. 21, 631–639. 10.1016/j.cmi.2015.02.006 25700888 PMC4470780

[B38] Lommano E. Bertaiola L. Dupasquier C. Gern L. (2012). Infections and coinfections of questing *Ixodes ricinus* ticks by emerging zoonotic pathogens in Western Switzerland. Appl. Environ. Microbiol. 78, 4606–4612. 10.1128/AEM.07961-11 22522688 PMC3370488

[B39] Majlathova V. Majlath I. Derdakova M. Vichova B. Pet'ko B. (2006). *Borrelia lusitaniae* and green lizards (*Lacerta viridis*), Karst Region, Slovakia. Emerg. Infect. Dis. 12, 1895–1901. 10.3201/eid1212.060784 17326941 PMC3291370

[B40] Majláthová V. Majláth I. Hromada M. Tryjanowski P. Bona M. Antczak M. (2008). The role of the sand lizard (*Lacerta agilis*) in the transmission cycle of *Borrelia burgdorferi* sensu lato. Int. J. Med. Microbiol. 298, 161–167. 10.1016/j.ijmm.2008.03.00517702653

[B41] Mannelli A. Bertolotti L. Gern L. Gray J. (2012). Ecology of *Borrelia burgdorferi* sensu lato in Europe: transmission dynamics in multi-host systems, influence of molecular processes and effects of climate change. FEMS Microbiol. Rev. 36, 837–861. 10.1111/j.1574-6976.2011.00312.x 22091928

[B42] Marcutan I. D. Kalmar Z. Ionica A. M. D'Amico G. Mihalca A. D. Cozma V.. (2016). Spotted fever group rickettsiae in ticks of migratory birds in Romania. Parasit. Vectors 9, 294. 10.1186/s13071-016-1565-7 27207258 PMC4875720

[B43] Matei I. A. Kalmar Z. Magdas C. Magdas V. Toriay H. Dumitrache M. O.. (2015). *Anaplasma phagocytophilum* in questing *Ixodes ricinus* ticks from Romania. Ticks Tick Borne Dis. 6, 408–413. 10.1016/j.ttbdis.2015.03.010 25838178

[B44] Michelet L. Delannoy S. Devillers E. Umhang G. Aspan A. Juremalm M.. (2014). High-throughput screening of tick-borne pathogens in Europe. Front. Cell. Infect. Microbiol. 4:103. 10.3389/fcimb.2014.00103 25120960 PMC4114295

[B45] Mihalca A. D. Dumitrache M. O. Sándor A. D. Magdaş C. Oltean M. Györke A.. (2012b). Tick parasites of rodents in Romania: host preferences, community structure and geographical distribution. Parasit. Vectors 5:266. 10.1186/1756-3305-5-266 23171665 PMC3514150

[B46] Mihalca A. D. Gherman C. M. Magdaş C. Dumitrache M. O. Györke A. Sándor A. D.. (2012a). *Ixodes ricinus* is the dominant questing tick in forest habitats in Romania: the results from a countrywide dragging campaign. Exp. Appl. Acarol. 58, 175–182. 10.1007/s10493-012-9568-3 22547023

[B47] Moise I. Dumitru S. (2012). Identifying aridization vulnerability zones in Dobrogea using medalus indices. Ann. Univ. Craiova Agric. Montanol. Cadastre Ser. XLII-1, 394–399.

[B48] Moutailler S. Valiente Moro C. Vaumourin E. Michelet L. Tran F. H. Devillers E.. (2016). Co-infection of ticks: the rule rather than the exception. PLoS Negl. Trop. Dis. 10:e0004539. 10.1371/journal.pntd.0004539 26986203 PMC4795628

[B49] Nieto N. C. Foley J. E. (2009). Meta-analysis of coinfection and coexposure with *Borrelia burgdorferi* and *Anaplasma phagocytophilum* in humans, domestic animals, wildlife, and *Ixodes ricinus*-complex ticks. Vector Borne Zoonot. Dis. 9, 93–102. 10.1089/vbz.2008.0072 18789001

[B50] Norman A. F. Regnery R. Jameson P. Greene C. Krause D. C. (1995). Differentiation of Bartonella-like isolates at the species level by PCR-restriction fragment length polymorphism in the citrate synthase gene. J. Clin. Microbiol. 33, 1797–1803. 7545181 10.1128/jcm.33.7.1797-1803.1995 PMC228273

[B51] Pavel I. Miron L. Rǎileanu C. Macovei I. Tronciu C. Acatrinei D. (2014). Seasonal dynamics of ixodid ticks in Iaşi urban area. J. Sci. Pap. Vet. Med. Univ. Agric. Sci. Vet. Med. Iaşi 57, 135–139.

[B52] Perez-Eid C. (2007). Les tiques. Identification, Biologie, Importance Medicale et Veterinaire (In french). Paris: Editions Medicales Internationales

[B53] Potkonjak A. Kleinerman G. Gutiérrez R. Savić S. Vracar V. Nachum-Biala Y.. (2016). Occurrence of *Borrelia burgdorferi* sensu lato in *Ixodes ricinus* ticks with first identification of *Borrelia miyamotoi* in Vojvodina, Serbia. Vector Borne Zoonot. Dis. 16, 631–635. 10.1089/vbz.2016.2008 27574731

[B54] Puchhammer-Stockl E. Kunz C. Mandl C. W. Heinz F. X. (1995). Identification of tick-borne encephalitis virus ribonucleic acid in tick suspensions and in clinical specimens by a reverse transcription-nested polymerase chain reaction assay. Clin. Diagn. Virol. 4, 321–326. 15566853 10.1016/0928-0197(95)00022-4

[B55] Rauter C. Hartung T. (2005). Prevalence of *Borrelia burgdorferi* sensu lato genospecies in *Ixodes ricinus* ticks in Europe: a metaanalysis. Appl. Environ. Microbiol. 71, 7203–7216. 10.1128/AEM.71.11.7203-7216.2005 16269760 PMC1287732

[B56] Regnery R. L. Spruill C. L. Plikaytis B. D. (1991). Genotypic identification of rickettsiae and estimation of intraspecies sequence divergence for portions of two rickettsial genes. J. Bacteriol. 173, 1576–1589. 10.1128/jb.173.5.1576-1589.1991 1671856 PMC207306

[B57] Reis C. Cote M. Paul R. E. Bonnet S. (2011). Questing ticks in suburban forest are infected by at least six tick-borne pathogens. Vector Borne Zoonot. Dis. 11, 907–916. 10.1089/vbz.2010.0103 21158500

[B58] Richter D. Schlee D. B. Matuschka F. R. (2011). Reservoir competence of various rodents for the lyme disease Spirochete *Borrelia spielmanii*. Appl. Environ. Microbiol. 77, 3565–3570. 10.1128/AEM.00022-11 21460106 PMC3127595

[B59] Rizzoli A. Hauffe H. Carpi G. Vourc H. G. Neteler M. Rosa R. (2011). Lyme borreliosis in Europe. Euro Surveill. 16:19906. 21794218

[B60] Sándor A. Mărcuţan D. D'Amico G. Gherman C. Dumitrache M. Mihalca A. (2014). Do the ticks of birds at an important migratory hotspot reflect the seasonal dynamics of *Ixodes ricinus* at the migration initiation site? a case study in the danube delta. PLoS ONE 9:e89378. 10.1371/journal.pone.0089378 24586732 PMC3929702

[B61] Schouls L. M. Van De Pol I. Rijpkema S. G. Schot C. S. (1999). Detection and identification of Ehrlichia, *Borrelia burgdorferi* sensu lato, and Bartonella species in Dutch *Ixodes ricinus* ticks. J. Clin. Microbiol. 37, 2215–2222. 10364588 10.1128/jcm.37.7.2215-2222.1999 PMC85121

[B62] Schwaiger M. Cassinotti P. (2003). Development of a quantitative real-time RT-PCR assay with internal control for the laboratory detection of tick borne encephalitis virus (TBEV) RNA. J. Clin. Virol. 27, 136–145 10.1016/S1386-6532(02)00168-3 12829035

[B63] Soleng A. Kjelland V. (2013). *Borrelia burgdorferi* sensu lato and *Anaplasma phagocytophilum* in *Ixodes ricinus* ticks in Bronnoysund in northern Norway. Ticks Tick Borne Dis. 4, 218–221. 10.1016/j.ttbdis.2012.11.006 23333106

[B64] Subramanian G. Sekeyova Z. Raoult D. Mediannikov O. (2012). Multiple tick-associated bacteria in *Ixodes ricinus* from Slovakia. Ticks Tick Borne Dis. 3, 406–410. 10.1016/j.ttbdis.2012.10.001 23182274

[B65] Swanson S. J. Neitzel D. Reed K. D. Belongia E. A. (2006). Coinfections acquired from ixodes ticks. Clin. Microbiol. Rev. 19, 708–727. 10.1128/CMR.00011-06 17041141 PMC1592693

[B66] Taragel'ova V. Koci J. Hanincova K. Kurtenbach K. Derdakova M. Ogden N. H.. (2008). Blackbirds and song thrushes constitute a key reservoir of *Borrelia garinii*, the causative agent of borreliosis in Central Europe. Appl. Environ. Microbiol. 74, 1289–1293. 10.1128/AEM.01060-07 18156328 PMC2258561

[B67] Tijsse-Klasen E. Sprong H. Pandak N. (2013). Co-infection of *Borrelia burgdorferi* sensu lato and Rickettsia species in ticks and in an erythema migrans patient. Parasit. Vectors 6:347. 10.1186/1756-3305-6-347 24326096 PMC3878868

[B68] van Duijvendijk G. Coipan C. Wagemakers A. Fonville M. Ersöz J. Oei A.. (2016). Larvae of *Ixodes ricinus* transmit *Borrelia afzelii* and B. miyamotoi to vertebrate hosts. Parasit. Vectors 9:97. 10.1186/s13071-016-1389-5 26896940 PMC4761128

[B69] Vassallo M. Paul R. Perez-Eid C. (2000). Temporal distribution of the annual nymphal stock of *Ixodes ricinus* ticks. Exp. Appl. Acarol. 24, 941–949. 10.1023/A:1010669003887 11354621

[B70] Vayssier-Taussat M. Le Rhun D. Buffet J. P. Maaoui N. Galan M. Guivier E.. (2012). Candidatus neoehrlichia mikurensis in bank voles, France. Emerg. Infect. Dis. 18, 2063–2065. 10.3201/eid1812.120846 23171720 PMC3557860

[B71] Vennestrom J. Egholm H. Jensen P. M. (2008). Occurrence of multiple infections with different *Borrelia burgdorferi* genospecies in Danish *Ixodes ricinus* nymphs. Parasitol. Int. 57, 32–37. 10.1016/j.parint.2007.07.004 17804280

